# Impact of Cyberbullying on Academic Performance and Psychosocial Well-Being of Italian Students

**DOI:** 10.3390/children11080943

**Published:** 2024-08-05

**Authors:** Antonio Ragusa, Sandra Núñez-Rodríguez, Paulo Vaz, José Silva, Virginia Caliciotti, Jerónimo J. González-Bernal, Alfonso J. López-Rivero, Ema Petrillo, Manuela Gatto, Ana Isabel Obregón-Cuesta, Josefa González-Santos

**Affiliations:** 1 Department of Education, Rome Business School, 00196 Rome, Italy; ragusa@romebusinessschool.it (A.R.); dipetrillo@romebusinessschool.com (E.P.); 2 Department of Health Sciences, University of Burgos, 09001 Burgos, Spain; vcx1002@alu.ubu.es (V.C.); jejavier@ubu.es (J.J.G.-B.); mjgonzalez@ubu.es (J.G.-S.); 3 Research Centre in Digital Sevices (CISeD), Instituto Politécnico de Viseu, 3504-510 Viseu, Portugal; paulovaz@estgv.ipv.pt (P.V.); jsilva@estgv.ipv.pt (J.S.); 4 Department Computer Science, University of Pontifical of Salamanca, 37002 Salamanca, Spain; ajlopezri@upsa.es; 5 Department of Education, University of Almería, 04120 Almeria, Spain; mg730@inlumine.ual.es; 6 Department of Specific Didactics, University of Burgos, 09001 Burgos, Spain; aiobregon@ubu.es

**Keywords:** cyberbullying, adolescents, psychosocial well-being, academic performance, Italy

## Abstract

Cyberbullying is a growing problem in the Italian educational sector, with a prevalence of 17%. This study analyzes its impact on the psychosocial well-being and academic performance of Italian adolescents. Method: A cross-sectional study was conducted with 502 students from six schools in different Italian regions, using the European Cyberbullying Intervention Project Questionnaire (ECIPQ) to assess cyberbullying, in addition to collecting data on satisfaction, friends, and academic performance. Chi-square and ANOVA analyses were conducted to identify significant associations between the variables. Results: The analyses showed significant associations between cyberbullying and gender and in psychosocial well-being, with significant differences in personal satisfaction and body satisfaction. On the other hand, there were no significant differences in academic performance or in the ability to make new friends, although victims showed a significantly lower ability to make new friends compared to those who were neither victims nor aggressors. Conclusions: Cyberbullying has a significant impact on students’ psychosocial well-being, especially on personal satisfaction and school happiness, making it essential to implement interventions that promote safe school environments to mitigate these negative effects.

## 1. Introduction

The phenomenon of cyberbullying has emerged in the last decade as a significant concern in the educational field due to the increased use of digital technologies among adolescents [1]. Globally, the prevalence of cyberbullying ranges from 10% to 40% among adolescents, highlighting the widespread nature of this issue [2]. In Europe, approximately 21% of adolescents have reported experiencing cyberbullying at some point [3], with variations across different countries influenced by factors such as internet usage rates and cultural attitudes towards bullying. Specifically, the prevalence of cyberbullying in Italy has reached 17% [4].

Cyberbullying, defined as harassment through digital means such as social networks, text messages, and emails [5,6,7,8], presents unique characteristics that differentiate it from traditional bullying. These differences include the ability of perpetrators to remain anonymous [9], the potential unlimited audience [9], and the possibility of constant harassment without temporal or spatial restrictions [10]. Risk factors for cyberbullying include age, gender, family relationships, and peer relationships, as well as cultural norms and societal values related to discrimination based on appearance, sexual orientation, ethnicity, or religion [11,12,13,14,15]. This phenomenon, which affects both victims and perpetrators, profoundly impacts the psychosocial well-being and academic performance of adolescents [16,17].

For victims, cyberbullying entails a form of harassment that is ubiquitous and persistent [10]. Unlike traditional bullying, which is usually confined to physical spaces and specific times, such as school, cyberbullying can follow victims to their homes, invading what should be their safe haven. Consequently, victims face a range of emotional and psychological problems, including anxiety, depression, low self-esteem, and even suicidal ideation [18,19,20]. These issues can lead to elevated levels of stress and symptoms of post-traumatic stress disorder (PTSD), comparable to those observed in victims of physical bullying [21,22]. Additionally, cyberbullying negatively affects interpersonal relationships and social adaptation in adolescents, increasing the risk of social isolation and problematic behaviors [23].

Research indicates that gender and age play significant roles in moderating the relationship between cyberbullying and psychological distress [20]. Girls are more likely to experience higher rates of cybervictimization and problematic social media use (PSMU) compared to boys [24,25]. This higher incidence among girls correlates with increased levels of emotional problems, such as anxiety and depression, whereas boys are more prone to externalizing behaviors [24,26]. On the other hand, the role that age plays in the psychosocial impact of cyberbullying may seem contradictory at first glance. Younger adolescents, particularly those around 11 years of age, typically receive higher levels of social support from family, friends, and teachers, which can buffer against the negative effects of cyberbullying, with this support diminishing as they grow older [20,27]. Moreover, despite this support, younger adolescents are still particularly vulnerable to the impacts of cyberbullying due to their developmental stage and less-developed coping mechanisms [20]. Additionally, cyberbullying is increasingly recognized as a crime as adolescents grow older [28].

These findings suggest a correlation between experiencing acts of harassment and lower life satisfaction, as well as the development of an unhealthy psychological identity [4,19]. Body image satisfaction, a specific component of general personal satisfaction, is significantly associated with online experiences [29]. Negative body image can lead to risky online behaviors, making adolescents more vulnerable to cyberbullying and its adverse effects. Studies have shown that body image issues can exacerbate the psychological impact of cyberbullying, leading to higher levels of depression, anxiety, and social withdrawal [29,30,31,32]. Addressing body image issues through education and supportive interventions can thus be crucial in reducing the impact of cyberbullying on adolescents’ psychosocial well-being. Moreover, adolescents who are both victims and perpetrators, often referred to as “bully–victims”, tend to experience even more severe psychological and social difficulties compared to those who are solely victims or perpetrators [33]. Bully–victims not only suffer from the emotional and psychological consequences of being targeted, but also from the guilt and internal conflict associated with their aggressive behavior, leading to complex emotional turmoil and a greater risk of long-term mental health issues. Additionally, these adolescents may struggle with forming and maintaining healthy relationships, exacerbating feelings of isolation and distress.

In addition to the impact on victims, perpetrators of cyberbullying also face negative consequences. Engaging in digital harassment behaviors can be associated with issues related to empathy and moral development, potentially leading to the internalization of aggressive and antisocial behaviors [16,17]. These adolescents often exhibit difficulties in their interpersonal relationships and may have a higher risk of engaging in other problematic behaviors, which also affects their psychosocial well-being and academic performance [33].

In academic terms, the impact of cyberbullying is profound, significantly affecting both victims and perpetrators. Academic performance encompasses not only grades but also school attendance, participation in academic activities, and overall educational achievements. Cyberbullying has been linked to a reduction in academic performance and increased absenteeism [34,35]. Victims of cyberbullying often experience a decline in their academic self-concept and mindfulness, which adversely affects their learning and social adaptation [36,37,38,39]. This deterioration is mediated by factors such as a sense of belonging and academic engagement, with observed differences between genders [40].

Conversely, students with lower academic performance are more likely to perpetrate cyberbullying compared to their peers with average or higher academic success. Cyberbullying is also negatively correlated with traditional protective factors, such as supportive family relationships, a positive school environment, and strong peer connections [41].

Schools play a crucial role in serving as a protective factor. They can mitigate the adverse effects of cyberbullying by fostering a safe and supportive environment, implementing effective anti-bullying policies, and promoting positive relationships among students and staff [42]. A positive school climate not only helps reduce the incidence of cyberbullying but also improves both academic and psychosocial outcomes for students [1].

Although previous research has provided valuable information on the impact of cyberbullying on adolescents, a more detailed examination of its specific effects on psychosocial well-being and academic performance in specific contexts is needed. This study aimed to analyze the impact of cyberbullying on these aspects among adolescents in various regions of Italy, contributing to a deeper understanding of the phenomenon. Specifically, we tested the following hypotheses:

1. There will be a significant relationship between gender and the categories of cyberbullying and bullying.
2. There will be a significant relationship between personal satisfaction and the categories of cyberbullying and bullying.
3. There will be a significant relationship between student happiness and the categories of cyberbullying and bullying.
4. There will be significant differences in students’ academic grades based on the categories of cyberbullying and bullying.

To address these hypotheses, our research question was: how does cyberbullying affect the psychosocial well-being and academic performance of adolescents in the Italian context? By addressing these hypotheses, our study aims to offer valuable insights for the development of effective interventions and policies to mitigate the adverse effects of cyberbullying on adolescents.

## 2. Materials and Methods

### 2.1. Desing of the Study

This study is framed as a multicenter, descriptive cross-sectional quantitative study conducted in various schools across different regions of Italy, aiming to analyze the impact of cyberbullying dynamics on academic performance and psychosocial well-being in students. The target population includes individuals aged 14 to 19 years, categorized into four roles: victim, perpetrator, both victim and perpetrator, and neither victim nor perpetrator.

### 2.2. Participants

The sample used in this research consisted of 502 secondary education students from six institutes located in different regions of Italy. The selected regions include Sicily (Catania), Campania (Naples), and Veneto (Verona). These regions were selected to capture a variety of cultural, economic, and social contexts, thus providing a broad and diverse basis for this study.

To ensure the representativeness of the sample, G* Power 3.1.9.7 software was used, calculating the need to recruit at least 180 participants to achieve a representative sample.

The sampling was conducted using a convenience sampling approach with elements of non-probabilistic stratified sampling. First, the participating institutes were selected based on the willingness and accessibility of directors and teachers to collaborate on this study. Subsequently, within each institute, a variety of educational profiles were sought to ensure the representation of students from different types of high schools (linguistic, scientific, classical, artistic, professional, and technical) and all levels of secondary education (from the first to the fifth year). This stratified non-probabilistic sampling strategy allowed us to capture the heterogeneity of the Italian educational system.

Regarding socio-demographic backgrounds, the sample was balanced in terms of gender, with 44.8% males and 55.2% females. The age of the students ranged from 14 to 19 years, with an average age of 16.65 years and a standard deviation of 1.44. This age distribution allowed us to examine how the studied factors affect students at different stages of their educational and personal development.

Additionally, the sample included a variety of types of institutes. The largest proportion of students came from classical institutes (47.8%, n = 240), followed by scientific high schools (13.7%, n = 69), and professional institutes (13.7%, n = 69). It also included students from artistic high schools (10.4%, n = 52), technical institutes (2.0%, n = 10), and linguistic high schools (1.6%, n = 8).

### 2.3. Procedure

For data collection, a questionnaire was administered to secondary school students from public centers. The administration of the questionnaire took place during school hours, with the permission of the school principal and the teacher in charge of the class at the time of administration. Prior to administering the questionnaire, students were informed about the purpose of the research, emphasizing that their participation was voluntary and their responses would be anonymous, and parental informed consent was obtained.

The data collection was conducted through self-reporting using the Google Forms platform to complete the questionnaire. Academic grades were collected via self-reporting, where students provided information on the grades they received for each subject during the previous semester. Additionally, students were asked to rate their happiness at school on a scale from 1 to 10. Overall satisfaction and satisfaction with physical appearance, as well as the recognition of cyberbullying as a perceived crime, were measured using a 5-point Likert scale, where 1 indicates “strongly disagree” and 5 indicates “strongly agree”. To evaluate the phenomenon of cyberbullying, the Spanish version of the European Cyberbullying Intervention Project Questionnaire (ECIPQ) [43] was used. This questionnaire consists of 22 Likert-type items with five response options, exploring both cybervictimization and cyberaggression. The ECIPQ has demonstrated good psychometric properties, with high reliability (Cronbach’s alpha = 0.88 for cybervictimization and 0.85 for cyberaggression) and strong construct validity, as evidenced by factor analyses in previous studies [43]. Based on their responses, students were categorized as a victim, aggressor, victim and aggressor, or neither victim nor aggressor, thus ensuring the representation of all roles within the phenomenon.

### 2.4. Statistical Analysis

The present study aimed to investigate the factors influencing cyberbullying in the academic and psychosocial domains of secondary school students in Italian institutes. For the data analysis, version 28 of the statistical software SPSS (IBM-Inc., Chicago, IL, USA) was used.

For inferential analyses, chi-square tests were employed to identify possible associations between various categorical variables. Significant differences between expected and observed frequencies were considered for absolute values exceeding 1.96 or less than −1.96 in the corrected residuals.

Additionally, to compare the means of continuous variables, such as academic grades and various indicators of psychosocial well-being (e.g., satisfaction with physical appearance, overall happiness, happiness at home, happiness at school, and happiness with friends) across different categories of cyberbullying, an analysis of variance (ANOVA) was used. Quantitative variables were subjected to the Kolmogorov–Smirnov test for normality, and although the data did not follow a normal distribution, parametric ANOVA tests were conducted, as studies have demonstrated the robustness of this test even in contexts with non-normal distributions [44].

## 3. Results

### 3.1. Association between Cyberbullying and Gender

Table 1 presents the results of the chi-square analysis conducted to investigate the relationship between gender and the categories of cyberbullying (neither victim nor aggressor, victim, aggressor, and victim and aggressor). A significant association was found in the distribution of these categories between boys and girls (χ^2^ (3) = 16.073, *p* = 0.001). These findings suggest that girls are more likely to be victims of cyberbullying compared to boys.

### 3.2. Association between Cyberbullying and Academic Performance

Table 2 presents the results of the analysis of variance (ANOVA) to determine the association between academic grades and cyberbullying. The results indicate that there are no significant differences in academic grades among the different categories of cyberbullying (*p* = 0.106).

### 3.3. Association between Cyberbullying and Personal Satisfaction

Table 3 presents the results of the ANOVA test to compare overall satisfaction with the categories of cyberbullying, showing significant differences with an F value of 2.97 and a *p* value of 0.031. This suggests that the level of overall satisfaction varies according to the degree of involvement in cyberbullying.

Additionally, post hoc tests revealed that individuals who were victims of cyberbullying had statistically lower overall satisfaction compared to those who were neither victims nor perpetrators (*p* = 0.012). Similarly, those who were both victims and perpetrators also showed significantly lower overall satisfaction compared to those who were neither victims nor perpetrators (*p* = 0.010).

### 3.4. Association between Cyberbullying and Satisfaction with Physical Appearance

Table 4 presents the results of the ANOVA used to compare satisfaction with physical appearance among different categories of students involved in cyberbullying, showing significant differences with an *F* value of 6.25 and a *p* value of 0.000. This indicates that at least one group differed significantly in their satisfaction with physical appearance compared to the other groups.

Further multiple comparisons revealed that victims had significantly lower satisfaction with their physical appearance compared to students who were neither victims nor aggressors (*p* = 0.005). Additionally, students who were both victims and aggressors also showed significantly lower satisfaction with their physical appearance compared to students who were neither victims nor aggressors (*p* = 0.000), with a mean of 3.34 compared to 3.92.

### 3.5. Asociation between Cyberbullying and School Happiness

Table 5 displays the results of the ANOVA test comparing school happiness among the different categories of cyberbullying. Post hoc tests revealed that victims had significantly lower levels of school happiness compared to students who were neither victims nor aggressors (*p* = 0.046). The mean happiness score for victims was 5.99, whereas for students who were neither victims nor aggressors, the mean happiness score was 6.50. Additionally, students who were both victims and aggressors exhibited significantly higher school happiness compared to victims (*p* = 0.019), with a mean of 6.59.

### 3.6. Asociation between Cyberbullying and Making New Friends

Table 6 presents the results of the ANOVA test to compare the ability to make new friends among students based on their experiences of cyberbullying. These results showed no significant differences, with an F value of 1.816 and a *p* value of 0.143. However, post hoc tests revealed that victims had a significantly lower ability to make new friends compared to those who were neither victims nor aggressors (*p* = 0.039).

## 4. Discussion

Due to the phenomenon of cyberbullying, significant concern has emerged in the educational sphere over the past decade as a result of the increased use of digital technologies among adolescent populations [1,4]. The findings of this study demonstrate the impact of cyberbullying dynamics on academic performance and psychosocial well-being in students. Consistent with previous literature, a significant trend was exhibited in gender participation in cyberbullying, indicating that girls are more likely to be victims compared to boys [40,43,45]. This can be attributed to multiple factors such as gender roles, social norms, and preferences for online communication [11,33,34,46,47].

Regarding the analysis of the impact of cyberbullying on academic performance, a lack of significant association between cyberbullying participation and academic grades was revealed. This finding contrasts with expectations, as cyberbullying is anticipated to negatively affect academic performance due to associated emotional stress and distraction. Although it is well-documented that bullying, including cyberbullying, can negatively impact mental health and academic performance [9,16,18,19], our results suggest that the direct impact on grades might not be as straightforward.

Several reasons might explain this lack of association. The Self-Determination Theory [48] suggests that students with high levels of autonomy, competence, and positive social relationships may be more resilient to the negative impacts of cyberbullying. Additionally, some students might develop effective coping strategies or seek appropriate support, buffering the detrimental effects on their academic performance.

However, the measures used to assess academic performance may not capture the full impact of cyberbullying on specific areas, such as motivation and classroom participation. Academic performance encompasses more than just grades, including school attendance and engagement. Further research is needed to explore these dimensions comprehensively.

Significant differences were observed in the impact of cyberbullying on students’ psychosocial well-being. It was found that cyberbullying victims experience lower levels of satisfaction with their physical appearance and overall well-being, as well as less happiness in school compared to those who are neither victims nor aggressors. Additionally, students who were both victims and aggressors, often referred to as “bully–victims”, also showed decreased satisfaction with their physical appearance and lower school happiness compared to victims and those not involved in bullying roles. The students with this dual role endure a heightened sense of insecurity and emotional stress due to their dual status. On one hand, they suffer from the direct impact of being targeted by cyberbullying, which adversely affects their self-perception and emotional well-being. On the other hand, engaging in bullying behaviors can lead to feelings of guilt and internal conflict, exacerbating their psychological distress. This combination of victimization and aggression intensifies emotional dysfunction, as they grapple with the negative effects of both experiencing and perpetrating cyberbullying. Furthermore, the dual role of a bully–victim can hinder their ability to form and maintain positive social relationships. The ambiguity in their self-image and the potential negative perceptions from peers can lead to decreased social interaction and support. These findings align with the existing literature [9,16,18,19].

It is noteworthy that cyberbullying victims have a significantly lower ability to make new friends compared to those who are neither victims nor aggressors. According to the existing literature, this finding may be attributed to decreased self-esteem and social confidence resulting from online harassment, which hinders the formation of new relationships [23,49]. As a phenomenon of persistent nature and with a potentially unlimited audience, cyberbullying could indeed cause a lasting impact on students’ social skills, affecting their ability to interact with peers and establish new friendships.

In light of these findings, practical implications should be emphasized to guide effective interventions against cyberbullying. Schools should implement comprehensive anti-cyberbullying programs, including training for staff and support mechanisms for affected students. Digital literacy should be integrated into the curriculum to help students manage online interactions safely. Additionally, fostering positive peer relationships and building students’ self-esteem through resilience programs can mitigate the psychosocial impacts of cyberbullying. Engaging parents through workshops on recognizing and addressing cyberbullying is also crucial for a collaborative approach.

The main limitation of this study lies in the data collection regarding students’ academic performance. Gathering academic grades from the previous year may not successfully reflect whether there was a significant variation in academic performance among those who experienced or perpetrated cyberbullying. Additionally, the statistical methods used may not capture the full complexity of the relationship between cyberbullying and academic performance. There is also a risk of bias in self-reported data due to social desirability or fear of repercussions. These limitations should be considered when interpreting the findings and assessing their applicability in different contexts.

Future lines of research should include longitudinal studies to track the evolution of cyberbullying and its long-term effects on students, providing a more comprehensive understanding over time. Additionally, it would be valuable to expand this study to include all roles in cyberbullying, including the roles of the reinforcer, bystander, and defender, investigating their implications on academic performance and psychosocial well-being.

## 5. Conclusions

The phenomenon of cyberbullying has raised significant concerns in the educational realm, especially with the increased use of digital technologies among adolescents over the past decade. This study reveals that, although no significant association was found between cyberbullying and academic performance, its impact on students’ psychosocial well-being is noteworthy. Victims of cyberbullying, as well as those who are both victims and aggressors, exhibit lower levels of satisfaction with their physical appearance and overall well-being and experience less happiness in the school environment. Additionally, victims face greater difficulties in making new friends due to decreased self-esteem and social confidence.

There is a higher likelihood for girls to be victims of cyberbullying compared to boys, due to factors such as gender roles and social norms. The primary limitation of this study lies in the sample size and the collection of academic grades from the previous year, which may not accurately reflect variations in academic performance.

These findings underscore the need for further research to promote safer and healthier school environments, where all students can develop fully and achieve their maximum potential.

## Figures and Tables

**Table 1:** Chi-square test to determine the association between cyberbullying and gender.

		Neither Victim Nor Aggressor	Victim	Aggressor	Victim and Aggressor
Boys	Count	89	41	15	80
	Expected Count	76.2	60.1	12.5	76.2
	Corrected residual	2.4	−3.9	1.0	0.7
Girls	Count	81	93	13	90
	Expected Count	93.8	73.9	15.5	93.8
	Corrected residual	−2.4	3.9	−1.0	−0.7

χ^2^ (3) = 16.073, *p* = 0.001.

**Table 2:** ANOVA test to determine the association between cyberbullying and academic performance.

	Academic Performance		ANOVA	
	Mean	Standard Deviation	F	Sig. (*p*)
Neither victim nor aggressor	80,193	13,192	2.054	0.106
Victim	84,430	126,449		
Aggressor	79,400	11,611		
Victim and aggressor	81,895	10,491		

**Table 3:** ANOVA test to determine the association between cyberbullying and personal satisfaction.

	Personal Satisfaction		ANOVA	
	Mean	Standard Deviation	F	Sig. (*p*)
Neither victim nor aggressor	4.11	0.871	2.977	0.031
Victim	3.82	1.03		
Aggressor	3.93	0.900		
Victim and aggressor	3.93	1.049		

**Table 4:** ANOVA test to determine the association between cyberbullying and body satisfaction.

	Body Satisfaction		ANOVA	
	Mean	Standard Deviation	F	Sig. (*p*)
Neither victim nor aggressor	3.92	1.16	6.25	0.000
Victim	3.51	1.37		
Aggressor	3.64	1.31		
Victim and aggressor	3.34	1.21		

**Table 5:** ANOVA test to determine the association between cyberbullying and school happiness.

	School Happiness		ANOVA	
	Mean	Standard Deviation	F	Sig. (*p*)
Neither victim nor aggressor	6.50	2.05	2.65	0.048
Victim	5.99	2.25		
Aggressor	5.82	2.58		
Victim and aggressor	6.59	2.27		

**Table 6:** ANOVA test to determine the association between cyberbullying and the ability to make new friends.

	Ability to Make New Friends		ANOVA	
	Mean	Standard Deviation	F	Sig. (*p*)
Neither victim nor aggressor	4.27	0.915	1.81	0.143
Victim	4.03	1.150		
Aggressor	4.36	0.911		
Victim and aggressor	4.17	0.948		

## Data Availability

https://docs.google.com/spreadsheets/d/15eSM5GMnwKdh8OL5sDZTJLmKzO8eKygv/edit?usp=sharing&ouid=106044932312011062063&rtpof=true&sd=true, accessed on 8 June 2024.
